# A decision tree built with parameters obtained by computed tomographic pulmonary angiography is useful for predicting adverse outcomes in non-high-risk acute pulmonary embolism patients

**DOI:** 10.1186/s12931-019-1160-5

**Published:** 2019-08-19

**Authors:** Dong Jia, Xue-lian Li, Qin Zhang, Gang Hou, Xiao-ming Zhou, Jian Kang

**Affiliations:** 1 Department of Emergency, Shengjing Hospital of China Medical University, No. 36, Sanhao Street, Shenyang, China; 2 Department of Epidemiology, School of Public Health, China Medical University, No.77, Puhe Road, Shenyang, China; 3 Department of Pulmonary and Critical Care Medicine, First Hospital of China Medical University, No.155, Nanjing North Street, Shenyang, 110001 China; 4 Department of Pulmonary and Critical Care Medicine, Shengjing Hospital of China Medical University, No. 36, Sanhao Street, Shenyang, 110004 China

**Keywords:** Computed tomographic pulmonary angiography, Pulmonary embolism, Pulmonary artery

## Abstract

**Background:**

Acute pulmonary embolism (APE) is one of the leading causes of death in cardiovascular disease. The 30-day mortality can still be 1.7–15% in non-high-risk APE patients. Some non-high-risk patients can progress into the high-risk group and even die, which is referred to as an adverse outcome. Promoting the diagnosis and predictive ability of adverse short-term prognosis was still a problem that needed to be solved. Computed tomography pulmonary angiography (CTPA) may be a way to promote the predictive ability. Our aim to develop predictive tools based on parameters obtained by computed tomographic pulmonary angiography (CTPA) in the form of a decision tree for use in non-high-risk acute pulmonary embolism (APE) patients.

**Methods:**

Adverse outcome was defined within 30 days after admission to the hospital. A decision tree was built to predict adverse outcomes based on discriminating factors screened from cardiac volume and clot characteristics from recursive partitioning analysis and compared with simplified pulmonary embolism severity index (sPESI), Bova scores and risk stratification. The area under the receiver operating characteristic curve (ROC-AUC) was used to confirm the predictive ability.

**Results:**

A total of 38 patients with and 303 patients without adverse outcomes were enrolled. Right ventricular/left ventricular (RV/LV) volume ratio, central pulmonary artery (CPA) embolism and right atria/left atria (RA/LA) volume ratio were used as splits in the decision tree to predict adverse outcomes in all patients. The ROC-AUC was 0.858. In CPA embolism patients, a recursive partitioning analysis was performed with cardiac volume and novel clot burden, but only the obstructing area (OA) ratio was included as a discriminating factor to build a second decision tree. The ROC-AUC for the second decision tree was 0.810. The decision trees were superior to those of sPESI, Bova scores and risk stratification, and there were no significant differences between the two decision trees.

**Conclusions:**

A decision tree built by CTPA parameters can predict adverse outcomes in non-high-risk APE patients.

## Introduction

Acute pulmonary embolism (APE) is one of the leading causes of death in cardiovascular disease [1, 2]. Differentiating high-risk APE patients from others on the basis of hypotension [1] and treating this group of patients with reperfusion therapy to save their lives are crucial due to the greater than 50% mortality rate in this group of patients [3]. However, these steps are not sufficient, as the 30-day mortality can still be 1.7–15% in non-high-risk APE patients [4]. Some non-high-risk patients can progress into the high-risk group and even die [1, 5], which is referred to as an adverse outcome [6]. Predicting non-high-risk patients with the likelihood of adverse outcomes is paramount for selecting the appropriate treatment and decreasing the mortality of APE in the short-term.

Currently, risk stratification [1], simplified pulmonary embolism severity (sPESI) and Bova scores [7] are the three main ways to predict short-term prognosis in the non-high-risk group of APE patients. However, their predictive ability is limited: sPESI is a useful tool to identify low-risk patients; and Bova scores, which were designed to predict short-term prognosis in the non-high-risk group of patients, still missed the diagnosis of some patients with adverse short-term outcomes [7]. Thus, promoting the diagnosis and predictive ability of adverse short-term prognosis was still a problem that needs to be addressed. Computed tomography pulmonary angiography (CTPA) may be a way to promote the predictive ability. Based on the severity of right ventricular dysfunction under pulmonary hypertension (PH), cardiac volume analysis has been helpful for predicting prognosis [8, 9] by CTPA. This cardiac volumetric change leads to clot blockage, which can be evaluated by clot location and clot burden analyses. Clot location correlates with the short-term prognosis [10]. However, there is still no widely accepted method to evaluate the clot burden for predicting short-term prognosis [10, 11].

Based on the needs of promoting the predictive ability and the usefulness of CTPA in APE, we measured cardiac volumes and evaluated novel clot burden methods, which referred to coronary computed tomographic clot burden [12, 13]. With these parameters described above, we developed decision trees and provided visual and simplified prediction models [14] for predicting short-term prognosis in the non-high-risk group of APE patients.

## Materials and methods

### Patient selection

**Fig. 1:**
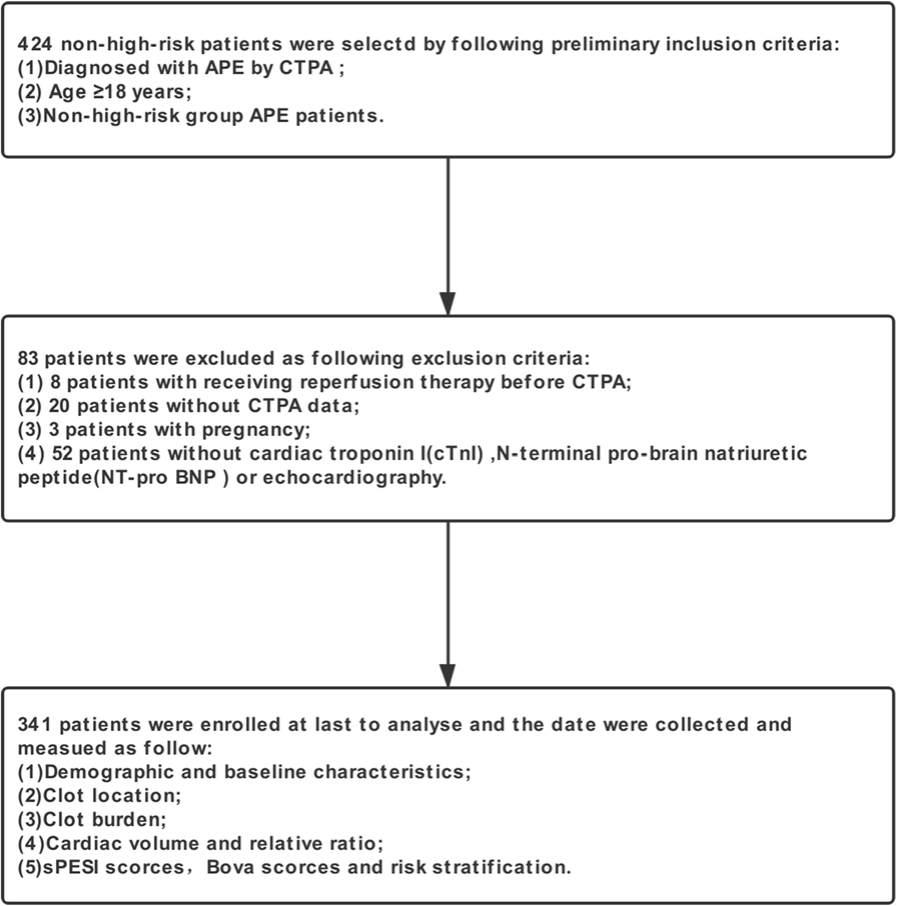
Flow chart of inclusion and exclusion criteria

This was a retrospective study from two research centers (First Hospital of China Medical University and Shengjing Hospital of China Medical University) in Shenyang, China. Between May 2014 to Dec 2018, 424 patients were collected from initially selected patients who were diagnosed with APE by CTPA, who were ≥ 18 years of age and non-high-risk APE patients (systolic blood pressure ≥ 90 mmHg, a systolic pressure drop by < 40 mmHg, or a systolic pressure drop ≥40 mmHg, but for ≤15 min) [1]. A total of 83 patients were excluded due to the following exclusion criteria: (1) 8 patients for receiving reperfusion therapy before CTPA; (2) 20 patients without CTPA data available for the reconstruction and evaluation of the clot analysis; (3) 3 patients who were pregnant; and (4) 52 patients without cardiac troponin I (cTnI), N-terminal pro-brain natriuretic peptide (NT- pro BNP) or echocardiography; ultimately, 341 patients were enrolled (Fig. 1).

### Clinical data

Demographic and baseline characteristics, heart rate, systolic pressure and past disease history were measured and defined from medical records upon admission to the hospital. Short-term prognosis was defined as adverse outcomes within 30 days after admission to the hospital. A positive adverse outcome (+) was defined as the occurrence of at least one of the following conditions: death; cardiopulmonary resuscitation; endotracheal intubation; vasopressor requirement for systemic hypotension (more than 5 μg per kilogram); or reperfusion treatment to save the patient’s life [6, 9]. These patients were grouped into the adverse outcome (+) group; otherwise, patients without adverse outcomes were identified and grouped into the adverse outcome (−) group.

### Risk stratification

All enrolled patients were divided into an intermediate–high-risk group, an intermediate-low-risk group and a low-risk group by right ventricular dysfunction (RVD), cTnI, and NT-proBNP [1]; RVD was confirmed by echocardiography [1]. cTnI>0.04 μg/L (normal range 0–0.04 μg/L) was defined as cTnI (+); otherwise, cTnI (−) was defined [15]. NT-proBNP (+) was defined as NT-proBNP ≥600 pg/mL; otherwise, NT-pro BNP (−) was defined [1].

### Calculation of the prediction score

The Bova [7] and sPESI [1] scores were calculated. The Bova score was converted into one of three classes (I-III). The sPESI score was converted into high- and low-risk patients.

### CTPA acquisition

CTPA was performed with an Aquilion KV-120 system (Toshiba Medical Systems Corporation, Tokyo, Japan) with a 64-detector row scanner. The parameters were set to 380 mA, 120 kV, and a 1-mm section thickness for reconstruction from the thoracic inlet to the upper abdomen. An iodinated nonionic solution (100 mL) was injected by an automatic dual-tube high-pressure injector (Ulrich REF XD 2051; Ulrich Medical GmbH, Ulm, Germany) into the antecubital vein at 4 mL/s.

### Central pulmonary artery reconstruction and segmentation

**Fig. 2:**
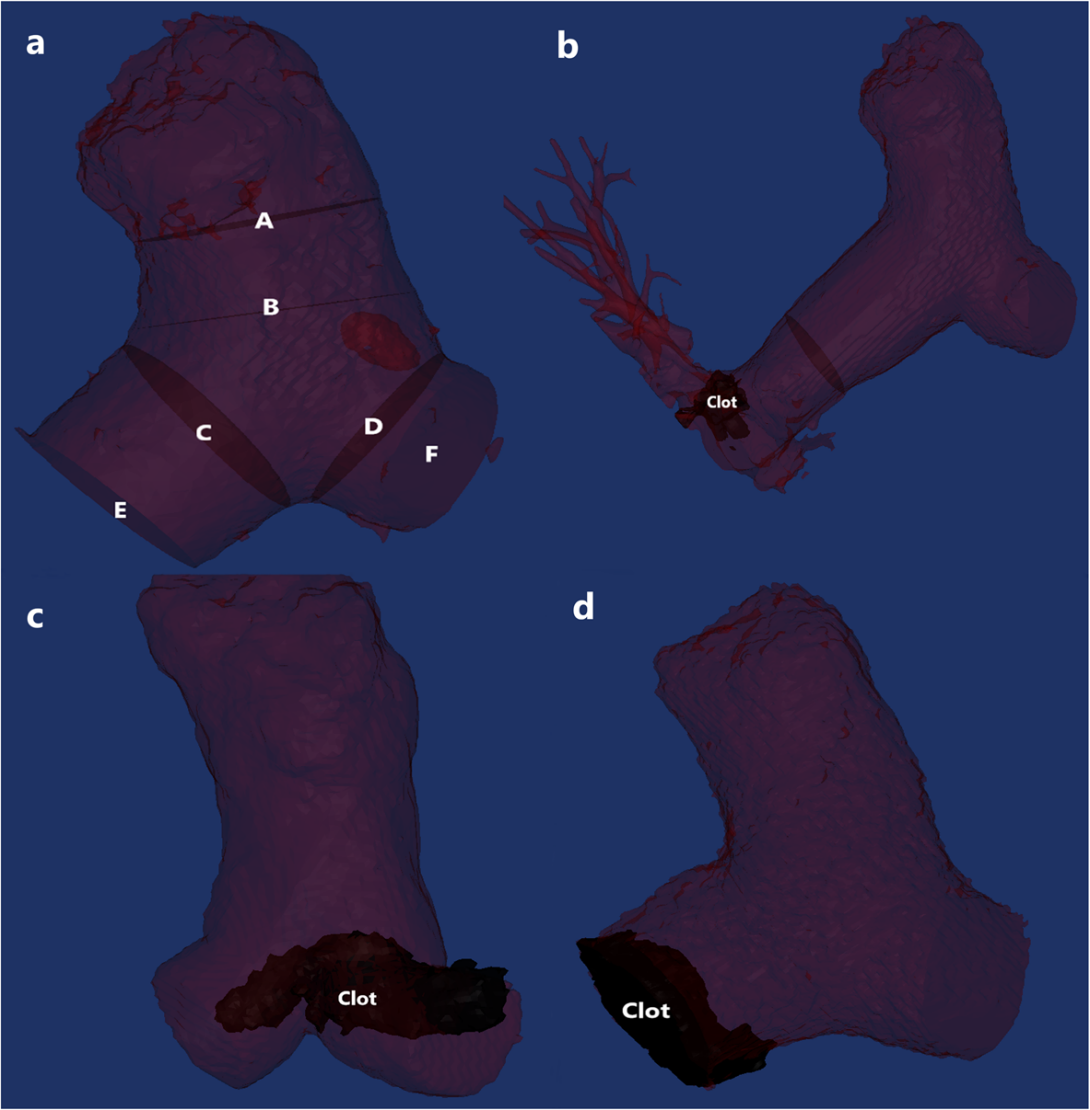
Central pulmonary artery reconstruction and selected plane. **a**. A, main pulmonary artery inlet plane; B main pulmonary artery outlet plane; C, right pulmonary artery inlet plane; D, left pulmonary artery inlet plane; E, right pulmonary artery outlet plane; F, left pulmonary artery outlet plane; The central pulmonary artery was reconstructed from the pulmonary artery inlet plane to right and left pulmonary artery outlet planes. **b**. Noncentral pulmonary artery embolism. **c**. Saddle-central pulmonary artery embolism. **d**. Central pulmonary artery embolism

All parameters were measured using Mimics Medical software (version 19.0, Mimics Medical software, Leuven, Belgium) and were recorded in the digital imaging and communications in medicine format. The central pulmonary artery (CPA) was reconstructed, including embolism from the main pulmonary artery (MPA) trunk inlet to outlets of the right and left pulmonary arteries (RPA and LPA). The centerline was established, and redundant lines were deleted manually based on the reconstructed CPA. Six center points were identified at six respective planes, including the MPA trunk inlet plane, the MPA trunk outlet plane, the LPA inlet plane, the LPA outlet plane, the RPA inlet plane and the RPA outlet plane on the reconstructed centerline. The segmenting CPA and selecting plane method refers to Schievano, S. et al. [16] and Barker, A. J.et al. [17]. The CPA was divided into four sections (MPA trunk, LPA section, RPA section and MPA triangular section) based on the centerline, six center points and selected planes mentioned above by an orthogonal cutting function (Fig. 2.a).

### Clot location

Clot locations were classified as follows: Saddle CPA embolism: embolism at the bifurcation location of the CPA [18]; CPA embolism: embolism at the CPA, including Saddle CPA embolism [19]; non-CPA embolism: embolism only at the segmental or sub segmental pulmonary artery (Fig. 2b, c, d).

### Clot burden evaluation

**Fig. 3:**
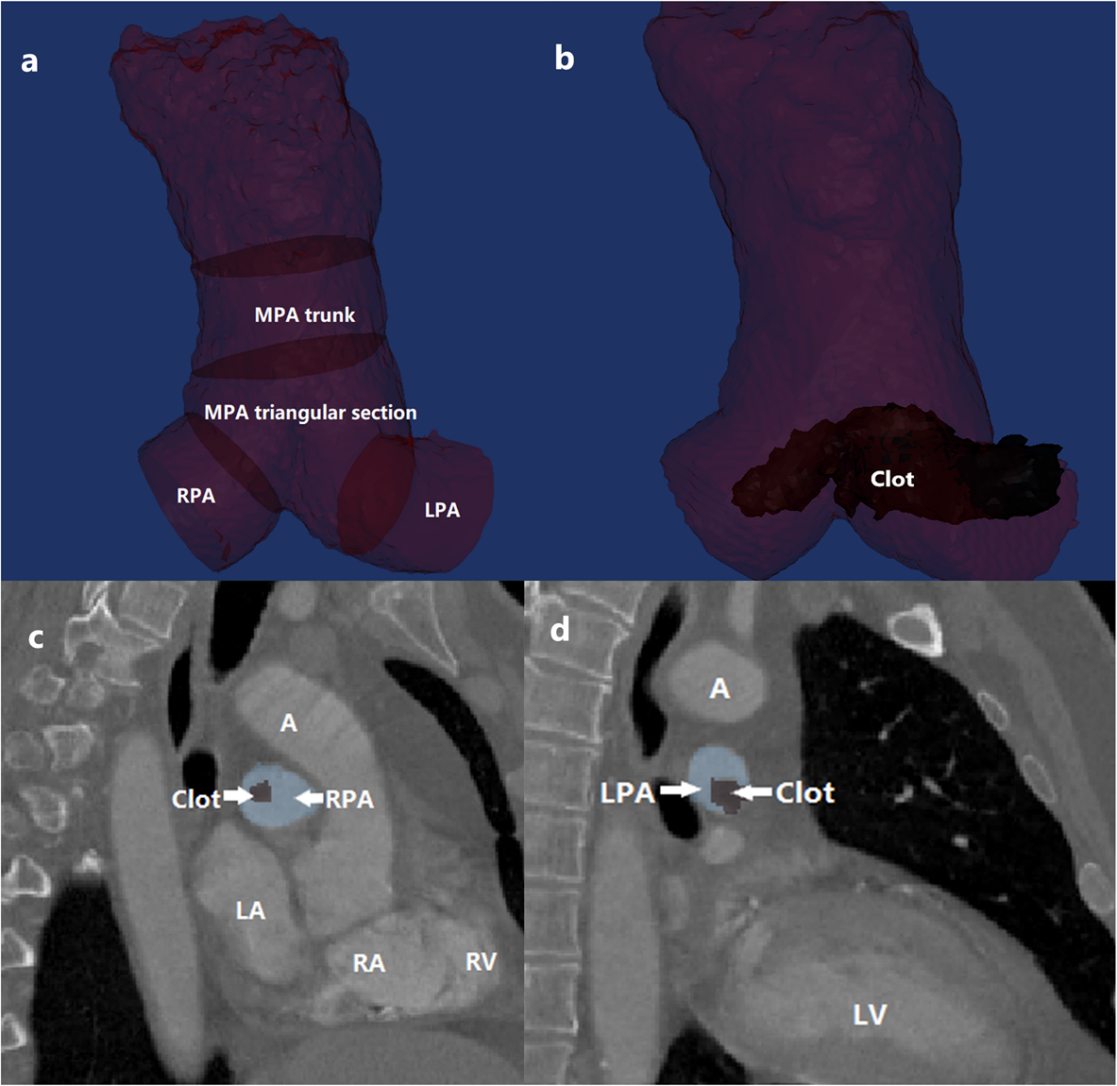
Obstruction area ratio. **a**. The central pulmonary artery was reconstructed into four sections: main pulmonary artery trunk, MPA triangular section, and left and right pulmonary arteries. **b**. The central pulmonary artery was reconstructed together with the embolism **c** and **d**. The maximal obstruction area ratio at the right and left pulmonary was calculated as half of the total maximal obstruction area ratio at the right pulmonary artery section and the left pulmonary artery section (A: aorta; RV: right ventricle; LV, left ventricle; RA, right atrium; LA, left atrium; LPA, left pulmonary artery; RPA, right pulmonary artery). The The obstruction area ratio was calculated as half of the total maximal obstruction area ratios perpendicular to long-axis of right and left pulmonary arteries as **c** and **d**

In CPA embolism patients, clots were segmented from the reconstructed CPA. Clot burden was then evaluated by five methods: (1) the widest clot diameters orthogonal to the long axis of the RPA and LPA were measured, and the total of the widest diameters in the RPA and LPA was recorded as the clot maximal diameter [10]; (2) the widest clot areas orthogonal to the long axis of the RPA and LPA were measured, and the total of the widest areas in the RPA and LPA was recorded as the clot maximal area; (3) the clot lesion length was measured on the centerline relative to the lesion CPA [13]; (4) the clot aggressive volume ratio was calculated as the ratio of the clot volume relative to the volume of the pulmonary artery in CPA embolism patients [12, 13], (5) The maximal ratio of embolism area to the artery area of corresponding plane, was calculated at RPA and LPA perpendicular to long axis respectively, which represented the percentage of maximal obstruction of pulmonary artery. The obstruction area (OA) ratio was calculated as mean of the two maximal ratios at the RPA and LPA above mentioned in CPA embolism patients [12] (Fig. 3a, b, c, d).

### Measurement of cardiac volumes

**Fig. 4:**
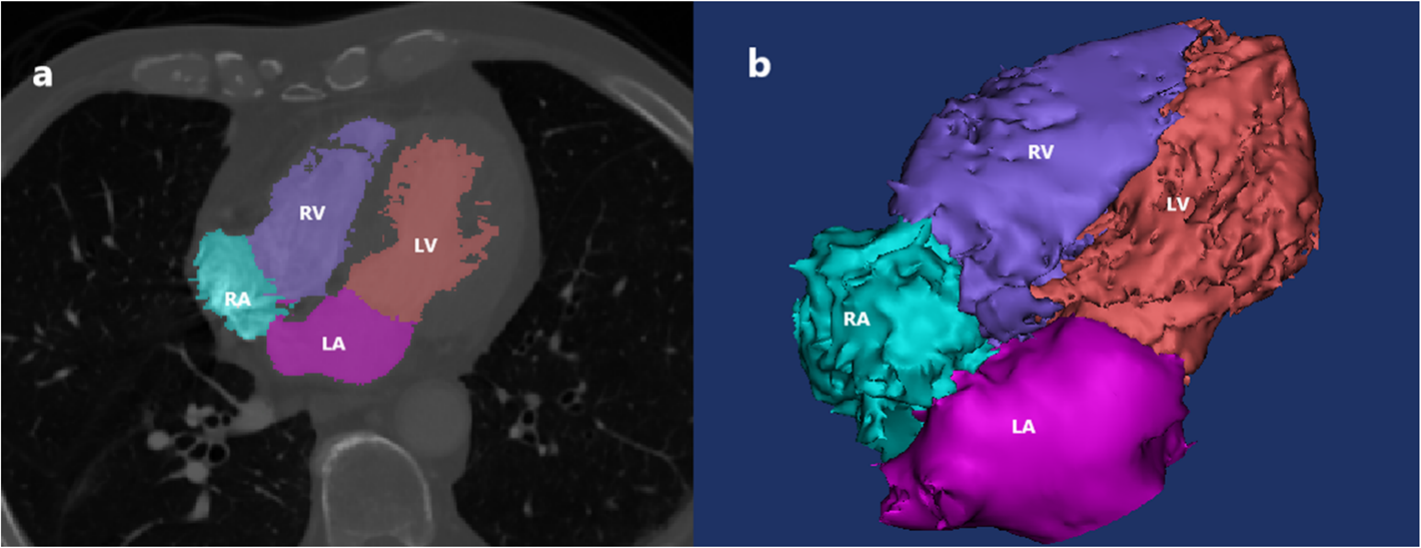
Cardiac volume measurement. **a**. The heart chambers were differentiated as RV (right ventricle), LV (left ventricle), RA (right atrium) and LA (left atrium). **b**. Every heart chamber was reconstructed, the volume parameters were measured, and the relative ratios were calculated

The cardiac chambers were segmented and reconstructed semi-automatically. The interatrial septum, interventricular septum and heart chamber walls were excluded, and the cardiac valves were used to segment different heart chambers and vessels. The cardiac capacity was measured according to the previous methods [8, 9]. The left atrial (LA) volume, right atrial (RA) volume, left ventricular (LV) volume and right ventricular (RV) volume were measured. The RA/LA and RV/LV volume ratios were calculated as the RA volume relative to the LA volume and the RV volume relative to the LV volume (Fig. 4a, b).

### Statistical analysis

Quantitative variables are expressed as the mean ± standard deviation (SD) and were analyzed by Student’s t-test to compare differences. Categorical variables are expressed as (+)/(−) and were analyzed by χ^2^ test to compare differences. A tree-based methodology was built to model predictive prognosis factors and to identify effect modifications with recursive partitioning analysis between different variables to determine which variables were identified less easily by other regression models [20]. Recursive partitioning analysis was used to evaluate all possible dichotomous splits for all potential decisional factors and choose the splits providing the optimal separations by binomial data [8, 21]. After each separation, the process was applied to each subgroup recursively until the subgroups reached a minimum size or no improvement could be made [22]. After pruning, a simple model was selected for clinical practice. Area under the receiver operating characteristics curve (ROC-AUC) analysis was used to evaluate the predictive ability of the decision tree predictive model. Predictive abilities were compared with the difference in ROC-AUC [23, 24]. A two-tailed *p* value less than 0.05 was considered to indicate a significant difference. The statistical analysis was performed with R software version 3.3.2 (http://www.R-project.org) and Medical statistical software (version 15.8, Belgium).

## Results

### Demographics, baseline characteristics and comparisons of the measured parameters in all the enrolled patients

Ultimately, 341 patients were enrolled. A total of 38 patients were defined as adverse outcome (+), and 303 patients were defined as adverse outcome (−). The RV volume, RA/LA volume ratio, RV/LV volume ratio, and the ratio of CPA embolism (+) to saddle CPA embolism (+) in the adverse outcome (+) group were higher than those in the adverse outcome (−) group, with significance (*p* < 0.001). LA and LV volumes in the adverse outcome (+) group were lower than those in the adverse outcome (−) group, with significance (*p* < 0.001) (Table 1).

**Table 1:** Comparison of parameters between adverse outcome (+) group and adverse outcome (−) patients in all enrolled patients (mean ± SD)

	Adverse outcome (+) group (*n* = 38)	Adverse outcome (−) group (*n* = 303)	*p* value
Age (year)	61.34 ± 12.72	60.60 ± 14.90	0.770
Sex (male/female)	18/20	136/167	0.936
RA volume (ml)	57.68 ± 14.97	54.69 ± 15.45	0.251
LA volume (ml)	44.46 ± 14.16	59.69 ± 13.85	< 0.001
RV volume (ml)	64.86 ± 21.58	51.05 ± 16.57	< 0.001
LV volume (ml)	51.71 ± 12.25	63.57 ± 9.93	< 0.001
RA/LA volume ratio	1.50 ± 0.82	0.97 ± 0.41	< 0.001
RV/LV volume ratio	1.34 ± 0.66	0.82 ± 0.30	< 0.001
CPA embolism/non/CPA embolism	31/7	103/200	< 0.001
Saddle CPA embolism/non-saddle CPA embolism	9/29	10/293	< 0.001

*RA* right atria; *LA* left atria; *RV* right ventricular; *LV* left ventricular; *CPA* central pulmonary artery

### Risk stratification, Bova scores and different classes of s PESI scores

Risk stratification: The ratio of adverse outcomes in the intermediate–high-risk group was 40.8%; the ratio of adverse outcomes in the intermediate–low-risk group was 13.0%, and the ratio of adverse outcomes in the low-risk group was 4.1% (Table 2).

**Table 2:** Comparison of parameters between different risk stratification groups (mean ± SD)

Parameter	Intermediate–high-risk group (*n* = 69)	Intermediate–low-risk group (*n* = 77)	Low-risk group (*n* = 195)
Age (year)	62.23 ± 12.20	62.42 ± 14.55	59.46 ± 15.43
Sex (male/female)	33/36	35/42	87/106
Adverse outcome (+)/(−)	20/49	10/67	8/187

Bova scores: The ratio of adverse outcomes in stage III was 44.4%; the ratio of adverse outcomes in stage II was 23.5%, and the ratio of adverse outcomes in stage I was 5.3% (Table 3).

**Table 3:** Comparison of parameters between different stages of Bova scores (mean ± SD)

Parameter	Stage III (n = 27)	Stage II (*n* = 51)	Stage I (*n* = 263)
Age (year)	62.19 ± 11.38	62.45 ± 12.93	60.19 ± 15.26
Sex (male/female)	15/12	24/27	116/147
Adverse outcome (+)/ (−)	12/15	12/39	14/249

sPESI score: The ratio of adverse outcomes in the high-risk group was 13.9%; the ratio of adverse outcomes in the low-risk group was 2.6% (Table 4).

**Table 4:** Comparison of parameters between different sPESI scores (mean ± SD)

Parameter	High-risk (n = 303)	Low-risk (n = 38)
Age (year)	62.25 ± 14.49	56.21 ± 15.40
Sex (male/female)	133/170	22/16
Adverse outcome (+)/(−)	37/266	1/37

### Demographics, baseline characteristics and comparisons of the measured parameters in CPA embolism patients

The RV volume, RA/LA volume ratio, RV/LV volume ratio, OA ratio, clot aggressive volume ratio, clot aggressive volume ratio, clot maximal diameter and clot maximal area in the adverse outcome (+) group were higher than those in the adverse outcome (−) group, with significance (*p* < 0.001, < 0.001, < 0.001, < 0.001, 0.001, < 0.001, 0.036 and < 0.001, respectively). The LA and LV volumes in the adverse outcome (+) group were lower than those in the adverse outcome (−) group, with significance (*p* < 0.001 and < 0.001, respectively) (Table 5).

**Table 5:** Comparison of parameters between adverse outcome (+) group and adverse outcome (−) patients in CPE patients (mean ± SD)

	Adverse outcome (+) group (*n* = 31)	Adverse outcome (−) group (*n* = 103)	*p* value
Age (year)	62.42 ± 11.16	59.84 ± 13.36	0.330
Sex (male/female)	14/17	47/56	0.963
RA volume (ml)	58.72 ± 15.15	54.24 ± 12.96	0.143
LA volume (ml)	43.18 ± 13.17	57.45 ± 17.65	< 0.001
RV volume (ml)	67.85 ± 22.16	52.67 ± 12.55	< 0.001
LV volume (ml)	50.80 ± 8.96	62.89 ± 9.72	< 0.001
RA/LA volume ratio	1.56 ± 0.82	1.01 ± 0.34	< 0.001
RV/LV volume ratio	1.39 ± 0.67	0.85 ± 0.25	< 0.001
Obstruction area ratio	0.38 ± 0.23	0.11 ± 12	< 0.001
Clot aggressive volume ratio	0.33 ± 0.25	0.16 ± 0.15	0.001
Clot lesion length (mm)	41.09 ± 35.32	13.75 ± 22.45	< 0.001
Clot maximal diameter (mm)	35.24 ± 24.35	16.01 ± 15.37	0.036
Clot maximal area (mm^2^)	374.36 ± 383.67	78.49 ± 139.83	< 0.001

*RA* right atria; *LA* left atria; *RV* right ventricular; *LV* left ventricular

### Decision tree and predictive ability for adverse outcome

**Fig. 5:**
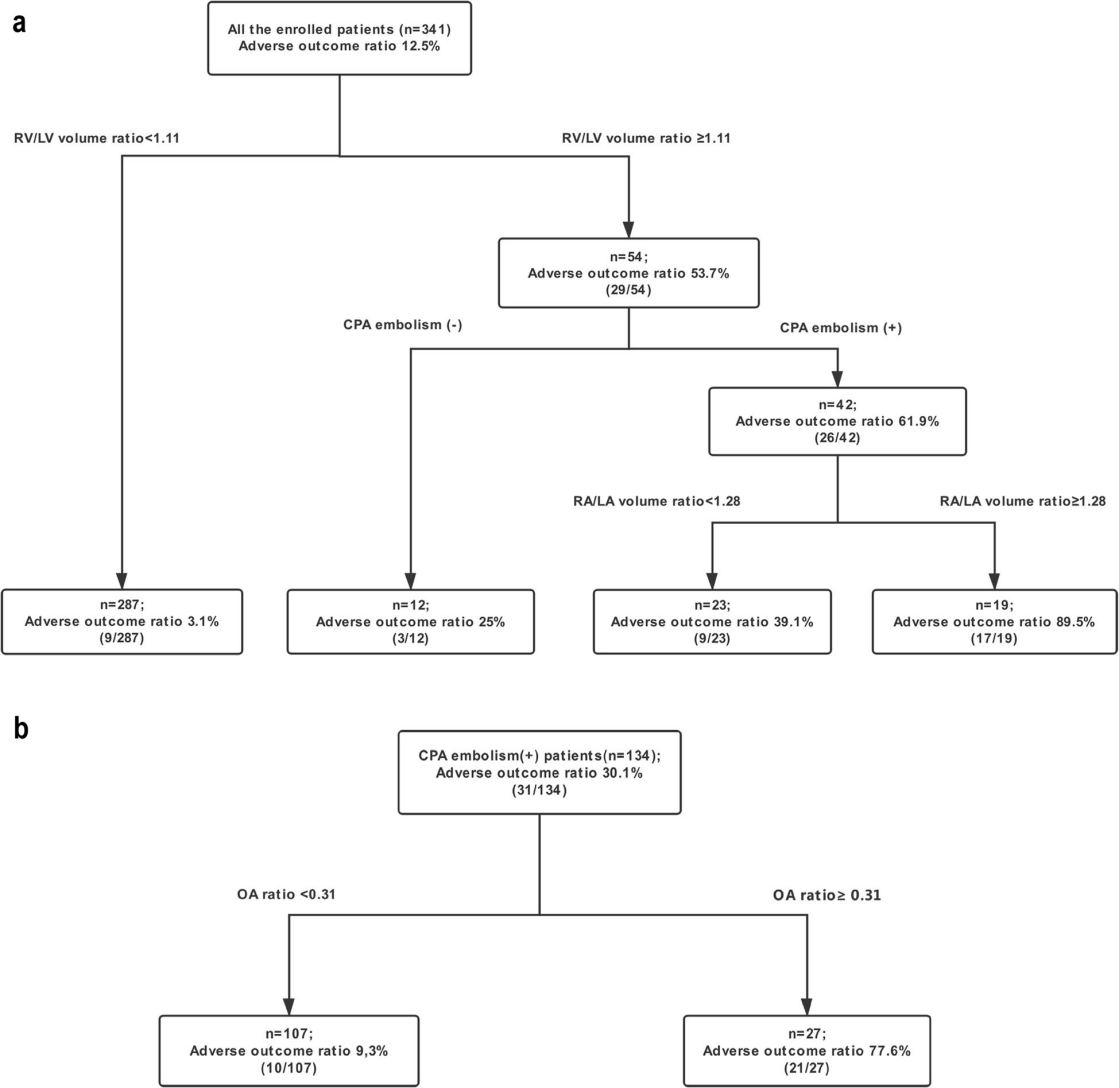
Decision trees for all patients and acute pulmonary embolism patients. **a**. The decision tree was developed by the RV/LV ratio, CPA embolism, and RA/LA volume ratio in the enrolled patients. **b**. OA ratio was optimal discriminating factor in CPA embolism patients

**Fig. 6:**
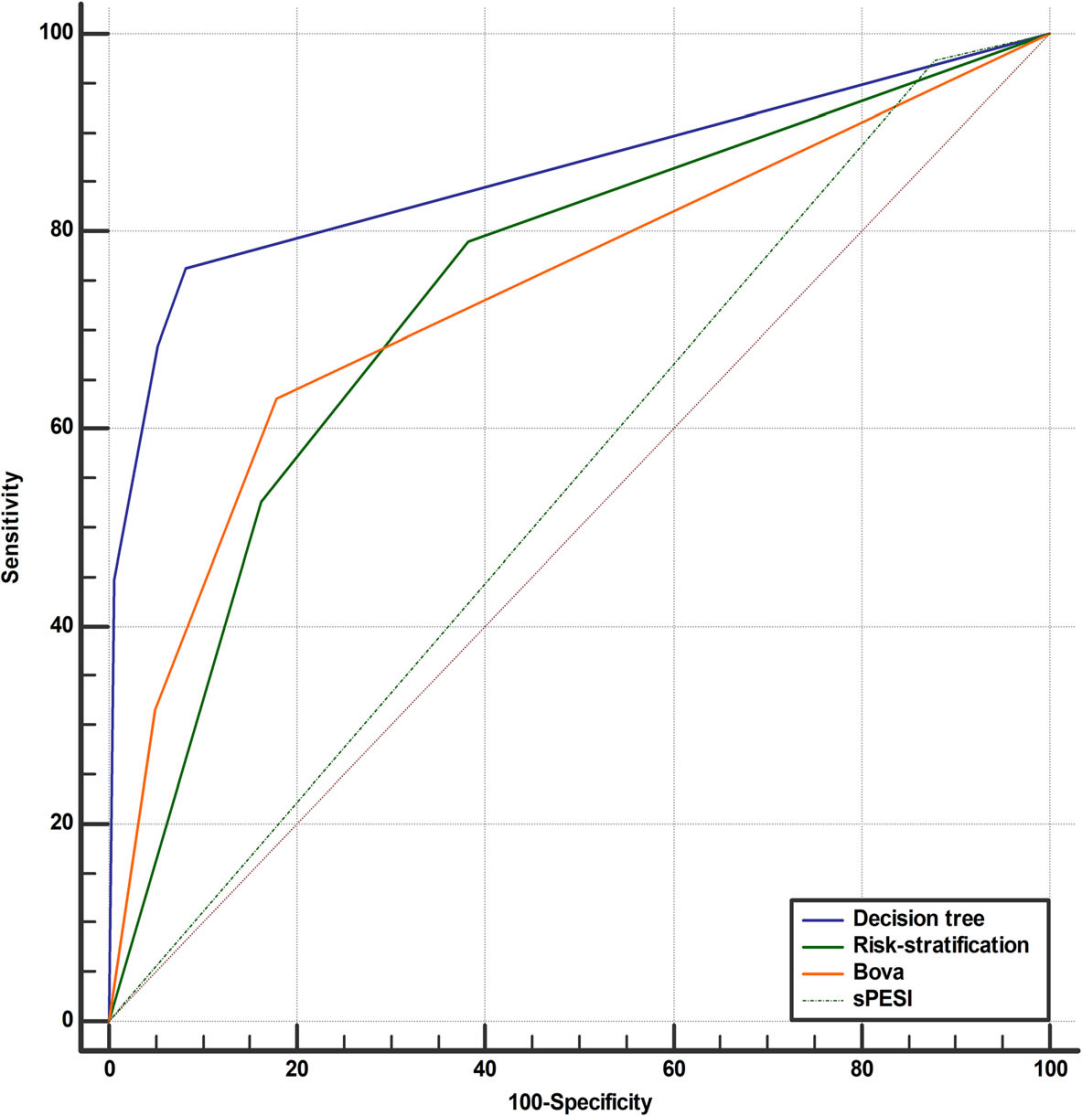
The predictive abilities of the decision tree and other predictive scores. The ROC curve built by the recursive partitioning analysis predictive model for all enrolled patients revealed that the area under the ROC was 0.858 (95%CI: 0.775–0.941). For all enrolled patients, the ROC-AUC revealed that the area of risk stratification, Bova scores and sPESI scores were 0.740 (95%CI: 0.690–0.786), 0.739 (95%CI: 0.689–0.785) and 0.548 (95%CI: 0.493–0.602), respectively. The predictive ability of the decision tree was better than others in the ROC-AUC (0.118, 95%CI: 0.0139–0.222, *p* < 0.05; 0.119, 95%CI: 0.00306–0.234, *p* < 0.05 and 0.310, 95%CI: 0.229–0.391, *p* < 0.05)

We developed a recursive partitioning model with cardiac volume and clot location together with sex and age. The decision tree was built based on recursive partitioning analysis. RV/LV volume ratio, CPA embolism and RA/LA volume ratio were included as discriminating factors in the decision tree for predicting adverse outcomes and formed four groups of relevance after three splits: (a) Split: all 341 enrolled patients were divided based on RV/LV volume ratio < 1.11 (*n* = 287) or ≥ 1.11 (*n* = 54), with adverse outcome ratios of 3.1 and 53.7%, respectively; (b) Split: patients were divided based on RV/LV volume ratio ≥ 1.11 patients with CPA embolism (+)/(−) (*n* = 42/12), with adverse outcome ratios of 61.9 and 25%, respectively; (c) Split: patients were divided based on RV/LV volume ratio ≥ 1.11 patients and CPA embolism (+) patients with RA/LA volume ratios < 1.28 (*n* = 23) or ≥ 1.28 (*n* = 19), with adverse outcome ratios of 39.1 and 89.5%, respectively (Fig. 5a). ROC-AUC built by the recursive partitioning analysis predictive model revealed that the area was 0.858 (95%CI: 0.775–0.941) (Fig. 6).

In CPA embolism patients, we also developed a recursive partitioning analysis with cardiac volume and novel clot burden methods together with sex and age. Only the OA ratio was included as a discriminating factor for predicting adverse outcomes. The recursive partitioning analysis formed two groups of relevance: Split: 134 CPA embolism patients were divided based on an OA ratio < 0.31 (*n* = 107) or ≥ 0.31 (*n* = 27), with adverse outcome ratios of 9.3 and 77.6%, (Fig. 5b). ROC-AUC revealed that the area was 0.810 (95%CI: 0.706–0.913).

### The difference in the predictive ability of the two decision trees

We compared the predictive abilities in ROC-AUC of the decision trees based on the volume index and on the OA ratio in CPA embolism. The difference was not significant (*p* = 0.48). We also compared the predictive ability between the decision tree and the risk stratification, Bova scores and sPESI scores (Fig. 6).

## Discussion

Predicting adverse outcomes in non-high-risk APE patients has been a significantly challenging problem [10]. Cardiac volume and relative ratio have also shown correlations with short-term prognosis in APE [8]. Clot blockage lead to this change of cardiac volume in substance. However, the method of evaluating severity of clot obstruction, called the clot burden, did not show a correlation with short-term prognosis in APE [1, 11]. Instead, clot location is a widely used applicable predictor for APE prognosis [10, 19]. Our result revealed that the RV/LV volume ratio was the first discriminating factor for predicting adverse outcomes with optimal differentiation degrees in the first decision tree of all APE patients. The RA/LA volume ratio was also a discriminating factor. The difference between the RV/LV and RA/LA volume ratios in terms of importance in the decision tree was caused by their different structures and reactions to pressure [8, 25]. Considering that the ratio between the right and left heart volumes has a relevance as a prognostic index, it would be interesting to know which is the main contribute to the increase of the ratio: right heart volumes enlargement or left heart volumes reduction. Our results showed that the left heart volumes reduction may be the main contribute to the adverse outcomes, as there was no difference in the RA volume between adverse outcome (+) group and adverse outcome (−) group whereas the LA volume of adverse outcome (+) group was smaller than that of adverse outcome (−) group. For the ventricular volume contributes, we deduced that LV contributed more than RV to the finial consequence of adverse outcome, because the hemodynamic collapses related to low cardiac output, was main manifestation of adverse outcome in APE patient. But, differentiating the weighing of the contribution of LV and RV to the RV/LV volume ratio was difficult, because the change of the RV and LV were dependent on each other and they were interactive due to the mutual compression. The decreased LV volume was led by the increasing RV stain, but when evaluated, RV and LV changed simultaneously. LV volume might be one of the discriminatory factors of the adverse outcome, but RV/LV volume ratio was the most optimal discriminatory factor when compared to single ventricular volume in the decision tree in our study. In addition, CPA embolism was another discriminating factor in the decision tree, which revealed the correlation between the cardiac volume and clot blockage. The decision tree integrated a module that could help the clinicians screen for the potential adverse outcome (+) patients.

CPA embolism correlates with prognosis [10], and it is an important discriminating factor for predicting adverse outcomes in the decision trees of our study. We evaluated the obstruction severity by half of the total maximal obstruction area ratios at the RPA and LPA sections in CPA embolism patients. In the subgroup of CPA embolism, we developed several novel methods for evaluating clot burden, including the maximal clot diameter, area, clot length, volume ratio and OA ratio, together with cardiac volume. The results showed that only the OA ratio was the optimal discriminating factor among these methods of clot burden in CPA embolism patients. The OA ratio was another way to identify adverse outcomes without measuring cardiac volume, which is a complicated process, and it could obtain a similar predictability as the decision tree built with the cardiac volume index and the CPA embolism.

Saddle CPA demonstrates embolism in bifurcation of the CPA and the clot blockage on the two sides of CPA. However, in our analysis, saddle CPA embolism was not a discriminating factor. The reason may be explained by the larger section at CPA bifurcation section than other sections [16], while the larger section represented a reduced influence on blood flow. The frequency of saddle CPA embolism was 5.6% in our study. However, our study may underestimate the occurrence probability of saddle CPA embolism and the associated mortality, because some patients with saddle CPA embolism with a poor prognosis and severe adverse outcomes would not survive before CTPA is performed, as indicated in a previous study [26].

Until now, traditional or classic methods of predicting the prognosis of APE have needed to be combined with clinical vital signs, such as echocardiography and biochemical indicators. As a diagnostic tool, CTPA only provides a static image, but it is still helpful for predicting adverse outcomes because relying solely on this static image is timesaving, as there is no need to wait for other results. In our study, the abilities for predicting the high probability of adverse outcome (+) patients and screening for adverse outcome (−) patients were superior than risk stratification and Bova scores. Discriminating using the decision trees built in our study would be easier, faster and more accurate than traditional methods. If only the cardiac volume index is available, or the CPA embolism is negative, the decision tree built by the integration of RV/LV volume ratio, CPA embolism and RA/LA volume ratio would be helpful in the discrimination. However, when the OA is available as the clot characteristics, while the patient is CPA embolism (+), the decision tree built with CPA embolism and OA would be easier than the cardiac volume index. Having PE diagnosis and prediction of PE severity in a single test is a potential advantage of using the decision trees built in our study.

The retrospective research design of our study limits the strength of our results. Additionally, our sample size was only 341. Different cardiac cycles could influence measurement cardiac volume. However, most studies describe measuring cardiac volume parameters with non-electrocardiographically gated computed tomographic angiography, including ours. The two decision trees in our study and the OA ratio, as a novel method for evaluating clot burden, still require further validation.

## Conclusion

Following the decision tree built using the RV/LV volume ratio, the RA/LA volume ratio and identifying a CPA embolism would facilitate the prediction of adverse outcomes in non-high-risk APE patients. In the CPA subgroup of patients, measuring the OA ratio is another way to predict adverse outcomes as a supplemental method.

## Data Availability

The datasets used and/or analyzed during the current study are available from the corresponding author on reasonable request.

## Abbreviations

APE: Acute pulmonary embolism
CPA: Central pulmonary artery
cTnI: Cardiac troponin I
CTPA: Computed tomographic pulmonary angiography
LA: Left atrial
LPA: Left pulmonary artery
LV: Left ventricular
MPA: Main pulmonary artery
NT-pro BNP: N- terminal pro- brain natriuretic peptide
OA: Obstruction area
PH: Pulmonary hypertension
RA: Right atrial
ROC-AUC: The area under the receiver operating characteristic curve
RPA: Right pulmonary artery
RV: Right ventricular
RVD: Right ventricular dysfunction
sPESI: Simplified pulmonary embolism severity index
